# Blood Lactate Is a Useful Indicator for the Medical Emergency Team

**DOI:** 10.1155/2016/5765202

**Published:** 2016-03-03

**Authors:** Maria Schollin-Borg, Pär Nordin, Henrik Zetterström, Joakim Johansson

**Affiliations:** ^1^Department of Anesthesiology and Intensive Care, Östersund Hospital, 83183 Östersund, Sweden; ^2^Department of Surgical and Perioperative Sciences, Umeå University, 90185 Umeå, Sweden; ^3^Department of Surgical Sciences, Anesthesiology and Critical Care Medicine, Uppsala University, 75185 Uppsala, Sweden; ^4^Department of Surgical and Perioperative Sciences, Anesthesiology and Intensive Care, Unit of Research, Education and Development-Östersund, Umeå University, 83183 Östersund, Sweden

## Abstract

Lactate has been thoroughly studied and found useful for stratification of patients with sepsis, in the Intensive Care Unit, and trauma care. However, little is known about lactate as a risk-stratification marker in the Medical Emergency Team- (MET-) call setting. We aimed to determine whether the arterial blood lactate level at the time of a MET-call is associated with increased 30-day mortality. This is an observational study on a prospectively gathered cohort at a regional secondary referral hospital. All MET-calls during the two-year study period were eligible. Beside blood lactate, age and vital signs were registered at the call. Among the 211 calls included, there were 64 deaths (30.3%). Median lactate concentration at the time of the MET-call was 1.82 mmol/L (IQR 1.16–2.7). We found differences between survivors and nonsurvivors for lactate and oxygen saturation, a trend for age, but no significant correlations between mortality and systolic blood pressure, respiratory rate, and heart rate. As compared to normal lactate (<2.44 mmol/L), OR for 30-day mortality was 3.54 (*p* < 0.0006) for lactate 2.44–5.0 mmol/L and 4.45 (*p* < 0.0016) for lactate > 5.0 mmol/L. The present results support that immediate measurement of blood lactate in MET call patients is a useful tool in the judgment of illness severity.

## 1. Introduction

The level and clearance ratio of blood lactate are well-known as useful parameters in the diagnosis and prognosis of the septic patient [1–4]. In a mixed Intensive Care Unit (ICU) setting, there is strong evidence for a correlation between high lactate levels and increased mortality [1, 5]. The dynamic progress of hyperlactatemia over the first 24 hours following ICU admission is a significant and independent determinator of illness severity in a mixed ICU-cohort [6] similar to findings in septic patients.

It has also been shown that lactate correlates with mortality in unselected patients at the emergency department (ED) [7] including older patients (>65 years) admitted with or without sepsis [8] and it was recently suggested to be included as a triage tool [9].

We could not find earlier studies where lactate levels have been studied in patients who are the subject of a Medical Emergency Team- (MET-) call.

The Medical Emergency Team is a concept first established in Australia around 1995 [10] and since then has spread to a large number of countries. The term used to describe the system varies and includes Rapid Response Team or Critical Care Outreach. There may be slight differences in how the system works, but the purpose is the same: early recognition of patients at risk, avoidance of ICU care if adequate, reduction of cardiac arrests, and reduction of in-hospital mortality.

The aim of the present study was to explore relations between blood lactate levels at the time of a MET-call and illness severity. We hypothesized that the blood lactate level was associated with an increased risk of 30-day mortality.

## 2. Material and Methods

### 2.1. Study Design and Setting

This is an observational study on a prospective and consecutively gathered cohort of MET-patients where concentration of arterial blood lactate at MET-calls during two years was registered. The study was performed at the regional secondary referral hospital of Östersund, Sweden, having a catchment area of approximately 126 000 inhabitants.

The hospital has 250 general beds with an annual turnover of approximately 20 000 patients, and a mixed ICU with 8 beds and 600 admissions per year. The overall in-hospital mortality is around 2%.

The study was approved by the Regional Ethics Review Board in Umeå.

The MET system has been employed at Östersund Hospital since 2007, covering all adult somatic wards. Our Medical Emergency Team consists of one intensive care physician and one intensive care nurse and is available 24 hours per day, seven days a week. The physician with primary responsibility for the patient and the nurse on the ward are also important members of the team assembled around the patient. The annual MET-call rate varies from 70 to 120. Our MET is not called to a cardiac arrest since there is another team responsible for cardiopulmonary resuscitation.

Any nursing staff on the ward can make the MET-call when predefined MET criteria are fulfilled, usually after contact with the physician responsible (though this is not a prerequisite). Our hospital uses a slightly modified version of the Early Warning Scoring (EWS) system, first described in 1997 [11]. Our criteria for a MET-call comprise any of the following: systolic blood pressure <90 mmHg, heart rate <40 or >130 beats/min, respiratory rate <8 or >30 breaths/min, peripheral oxygen saturation (SpO_2_) <90%, and sudden decrease in consciousness and/or intuition/serious concern for the patient. It is defined that we shall always see the patient within 30 minutes from the call.

### 2.2. Patients

All patients who were the subject of MET-call between March 2012 and March 2014 were screened for eligibility, regardless of age and comorbidity. Patients were excluded only if a blood lactate was unavailable.

### 2.3. Data Collection

Patient demographics (age, gender), vital signs (systolic blood pressure, heart rate, respiratory rate, and peripheral oxygen saturation), and the reason for the MET-call were collected from records kept at the time of the call. Lab results, comorbidity, the event of transfer to the ICU, therapy received (including vasoactive agents, mechanically assisted ventilation), length of hospital stay as well as ICU stay, and death within 30 days were collected from the medical records database. Blood lactate analysis was performed at our hospital laboratory using ABL 800 Flex Analyzer (Radiometer Medical, Copenhagen, Denmark. Test range 0.5 to 15 mmol/L). The reference range is 0.63–2.44 mmol/L and the normal time to get a result is about 15 minutes in total from when the sample is taken.

Primary outcome was 30-day mortality.

The cohort was stratified in different lactate intervals, roughly based on the quartiles, for analysis of a possible correlation between mortality and level of blood lactate.

To further explore the impact of hyperlactatemia, we divided the cohort in four groups based on presence of hyperlactatemia and acidosis. Mortality was calculated in these different groups.

### 2.4. Statistical Analysis

We divided the whole cohort of patients into two groups; survivors and nonsurvivors at 30 days from the MET-call.

The Mann-Whitney *U* test was used to compare numeric variables, such as lactate concentration, vital signs, and length of hospital stay between the groups. A two-tailed *p* value less than 0.05 was chosen as limit for significance, but, to compensate for multiple comparisons, the Bonferroni correction was used in the univariate analyses, giving a corrected level of significance *p* = 0.0083 (for the six different tests in univariate analysis). Data are presented as percentage, median, and interquartile range (IQR).

Odds Ratios (OR) for mortality in the groups with lactate 2.44–5.0 and ≥5.0 mmol/L were calculated with the group <2.44 as a reference.

Sensitivity, specificity, positive predictive value (PPV), and negative predictive value (NPV) for 30-day mortality were calculated for the two cut-off levels of 2.44 and 5.0. Confidence intervals were calculated with the Wilson score method.

We further analyzed the relationship between the independent variables age, blood lactate, and oxygen saturation (i.e., the parameters with *p* < 0.05 in the univariate analyses) and the outcome measure 30-day mortality in a multiple logistic regression.

Analyses were carried out using Statistica 12 (Statsoft®, Tulsa, OK, USA).

## 3. Results and Discussion 

**Figure 1:**
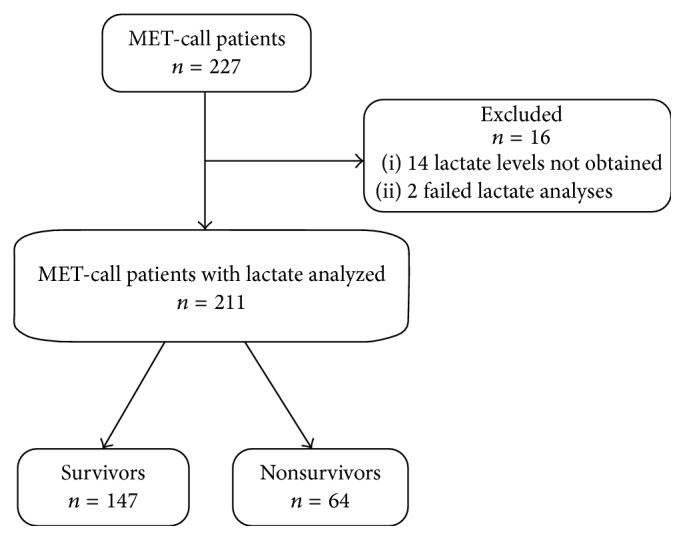
Enrolment and outcomes of study patients (MET: Medical Emergency Team).

During the study period 227 patients were the subject of MET-call and hence eligible for inclusion. Sixteen patients were primarily excluded for reasons described in Figure 1.

227 MET-call patients in this study reflect the usual calling rate for our hospital when compared to the years preceding. Ninety-three percent of cases had a lactate measurement result suggesting that our findings may be applied to MET-call patients in general. Age, gender, and referral ward are reported in Table 1.

The median age of our patients was high, 78 years, but this seems representative of MET-call patient cohorts described in the literature that are typically over 70 years of age [12]. It should be noted that MET-call patients have a high mortality rate, even when excluding patient assigned limitations of medical treatment. The mortality in the current study matches other similar cohorts [12, 13] but is higher than others [14]. The reason for different mortality in different settings is probably related to differences in how the system is used and built, for example, the attitude by ward personnel to MET-call a patient with limitations of medical treatment and the availability of other medical support (e.g., related to the work load for the medical, surgical, and orthopaedic physicians on call at the emergency department).

As can be seen in Table 1, the different wards used the MET-system to a varying degree as measured by MET-calls per 1000 admissions. There are likely multiple reasons for this. Department of medicine has, as opposed to the other departments in our hospital, access to a high dependency unit explaining the low frequency of MET-calls from the medical wards.

The most common reason for activation of the MET-call was decreased oxygen saturation (56%), followed by abnormal respiratory rate (30%), hypotension (28%), neurological derangement (21%), tachy-brady arrhythmias (16%), and intuition (15%). Many of the patients fulfilled 2 or more call criteria (54%) (note: registration of respiratory rate was missing in 25% of the MET-calls).

The patients had a wide variety of comorbidities, including diabetes, coronary artery disease, malignancy, infection, postorthopedic surgery, ileus, chronic obstructive pulmonary disease, congestive heart failure, and chronic renal insufficiency.

Median lactate concentration at the time of the MET-call was 1.82 mmol/L (IQR 1.16–2.7). The 30-day mortality was 30.3%, and in-hospital mortality was 28.4%. The median length of hospital stay was 13 days (IQR 7–24) (Table 1).

**Figure 2:**
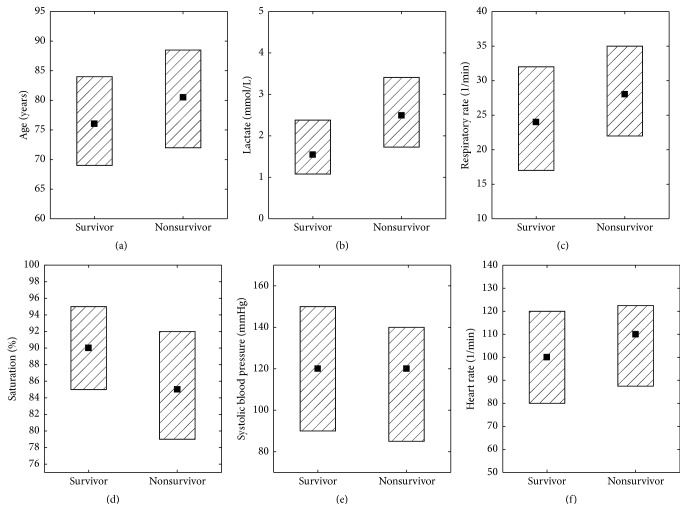
Age (a), lactate (b), respiratory rate (c), saturation (d), systolic blood pressure (e), and pulse (f) in relation to 30-day mortality, *n* = 211. Square and box indicate median and interquartile range.

When comparing the survivor and nonsurvivor groups in the cohort, including age, lactate, and vital signs, we found a statistically significant difference for lactate and oxygen saturation, a trend for age but no association with respiratory rate, heart rate, and systolic blood pressure (Table 2 and Figure 2). There were no significant gender differences (data not shown). The fact that age is correlated with mortality is not controversial.

**Figure 3:**
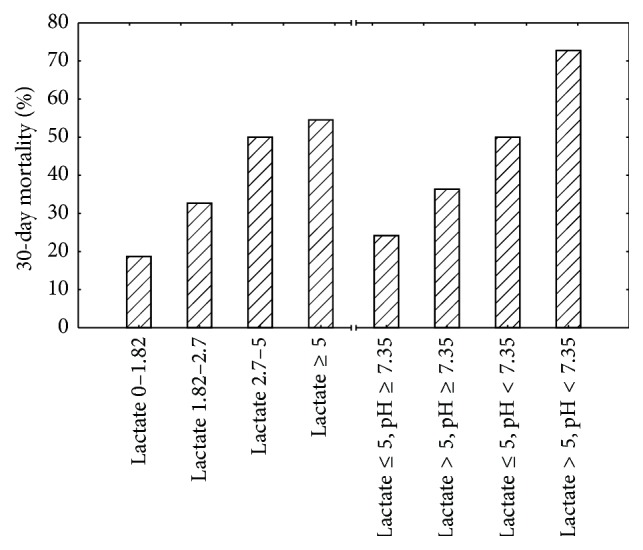
30-day mortality for different lactate intervals and levels of pH at Medical Emergency Team calls (*n* = 211).

To further illustrate the relationship between lactate at the time of a MET-call and mortality, we stratified the cohort into four groups based on the quartiles and one extreme group (0–1.81 (quartiles 1 and 2); 1.82–2.7 (quartile 3); 2.71–5.0 (lower part of quartile 4); and ≥5.0). We also divided the cohort into four different groups based on the presence of extreme hyperlactatemia (>5 mmol/L) and acidosis (pH < 7.35).  Figure 3 shows the respective 30-day mortality for each of these groups.

It is not surprising but well worth stating that whether or not the hyperlactatemia is associated with acidosis or not has implications for the risk of death (Figure 3). This illustrates that the patients that have the capacity to compensate for lactic acid with other systems are better off than the others.

The OR for 30-day mortality when the patients are stratified into 3 lactate levels is displayed in Table 3.

The accuracy of prediction of 30-day mortality with blood lactate (sensitivity, specificity, positive, and negative predictive value) based on the cut-off values 2.44 mmol/L and 5.0 mmol/L is presented in Table 4.

The sensitivity and PPV of high lactate (e.g., ≥5 mmol/L) may not be impressive (Table 4) but it is important to remember that the chosen endpoint is hard (30-day mortality). Any laboratory test would certainly fail to predict mortality with high accuracy in this setting. Even with the relatively low PPV, our results suggest that it is reasonable to transfer a patient subject to MET-call to the ICU if lactate is above 5, considering the nature of our endpoint. The mortality in this group of patients was 55% despite the fact that 11/22 patients were immediately transferred to the ICU. Out of the 11 not transferred, five were definitely the subject of limitations of medical treatment (recorded properly in their medical records) of which four died. Our data does not allow us to explore why the remaining six patients with lactate above 5 mmol/L were not transferred to the ICU but mortality was only 1/6. This suggests that the decision not to transfer probably was correct despite the high lactate and that hyperlactatemia may have been related to a relatively benign cause, such as a seizure that could be controlled. Sensitivity, specificity, and positive and negative predictive values will be highly dependent on the quality of resuscitation as well as on the attitude toward MET-calls for patients with limitations of medical treatment.

The other important results to be found in Table 4 are that the specificity and NPV for both cut-offs are fairly good. This can be related to, for example, the results of Barfod et al. [9], who recently found the same in a somewhat healthier cohort of patients at the emergency reception.

The multiple logistic regression analysis for the parameters age, lactate, and oxygen saturation as possible determinants of 30-day mortality revealed that lactate and oxygen saturation were significant independent variables but not age (Table 5).

Our results indicate that blood lactate may even be a better indicator of risk for death than traditional vital signs when it comes to MET patients. Ordinary patient monitoring may have the limitation that vital signs may not change until the patient reaches a critical stage [15]. Although patients appear hemodynamically stable they may have ongoing occult hypoperfusion and thereby increased blood lactate levels. Howell et al. have presented data, from patients admitted to hospital with clinically suspected infection, showing that a single blood lactate level provides important prognostic information that is even more accurate than hemodynamic status and comorbidity [16]. The SOCCER-study found that disturbed arterial blood gas was associated with in-hospital death but here this sigh was not a stronger predictor than clinical disturbances in respiration or circulation [17].

The relation between illness severity and lactate is not new but to our knowledge this is the first study to assess the relationship between blood lactate concentration and mortality in a consecutive cohort of MET patients. Jones et al. [18] recently suggested in an educational article that MET patients with a lactate > 3 mmol/L be transferred to the ICU.

Our study has several weaknesses. Registration of respiratory rate was missing in 25% of cases, reducing the value of respiratory rate as a predictor and thereby influencing our results to some degree. This vital sign is often forgotten and therefore not registered [19] which is unfortunate since an abnormal respiratory rate has been shown to be an important predictor of later deterioration in a cohort of patients at the emergency department [20] and of a serious event such as cardiac arrest [21].

Further, the design of the study is observational.

Some patients were the subject of limitations of medical treatment. This was often discovered in relation to the MET-call which is common [13]. We regret that it was impossible to identify these patients for a subgroup analysis due to the retrospective study design. We found that limitations of medical treatment (i) were not uniformly documented; (ii) sometimes were not documented despite it could be anticipated from the medical record; and (iii) could refer to one or several limitations of varying degrees of seriousness (no cardiopulmonary resuscitation, no ventilator treatment, palliation, and no ICU-care).

Our data indicate that the measurement of blood lactate is helpful in the assessment of a MET patient. In univariate analyses, an increased blood lactate was associated with higher mortality. In multiple regression, the only significantly independent parameters associated with mortality were lactate and oxygen saturation. An almost four times increased risk of mortality was shown for the MET patients with a blood lactate ≥ 5.0 mmol/L.

## 4. Conclusion

In conclusion, a high blood lactate concentration in MET-call patients was associated with higher 30-day mortality. In multiple regression, our data even show that blood lactate may be a better predictor of mortality than traditional vital signs. The results support the practice of routine measurement of blood lactate in MET-call patients.

## Figures and Tables

**Table 1:** Characteristics of patients subject to a Medical Emergency Team- (MET-) call.

Characteristic	Value, median (interquartile range)
Number of patients	211
Age, years	78 (69–84)
Gender, % female : % male	48 : 52
Lactate, mmol/L	1.82 (1.16–2.7)
Length of hospital stay, days	13 (7–24)
Length of ICU stay, days	3 (2–7)
Length of mechanically assisted ventilation, days	5 (7–24)
30-day mortality rate, %	30.3
In-hospital mortality rate, %	28.4
Referral ward, number of calls (number/1000 admissions)	
Surgical	64 (7.3)
Orthopedic	71 (13.2)
Medical	40 (3.1)
Infectious diseases	30 (16.7)
Gynecology	3 (0.7)
Ear, nose, and throat	1 (1.4)

**Table 2:** Characteristics of survivors and nonsurvivors after 30 days, univariate analysis.

	Survivors value, median	Nonsurvivors value, median	*p* value	*n* (%) of patients fulfilling criteria
Number of patients	147	64		
Age, years	76	81	0.0199	
Lactate, mmol/L	1.54	2.50	<0.001^*∗*^	
Respiratory rate, breaths/min	24	28	0.092	63 (30%)
Oxygen saturation, %	90	85	<0.002^*∗*^	119 (63%)
Systolic blood pressure, mmHg	120	120	0.374	59 (28%)
Heart rate, beats/min	100	110	0.237	33 (16%)

^*∗*^Significant tests (level of significance set at *p* = 0.0083 with respect to Bonferroni correction for multiple comparisons for the six different tests).

**Table 3:** Lactate categorised in three groups, OR for 30-day mortality.

Lactate (mmol/L)	*n*	OR	95% CI	*p*
≤2.44	146	Reference		
2.44–5.0	43	3.54	1.73–7.26	0.0006
≥5.0	22	4.45	1.76–11.26	0.0016

OR, Odds Ratio.

**Table 4:** Prediction of 30-day mortality with lactate cut off 2.44 and 5.0 mmol/L.

Cut-off 2.44 mmol/L			Cut-off 5.0 mmol/L		
	30-day mortality			30-day mortality	
	No	Yes		No	Yes
Lactate ≤ 2.44 mmol/L	115	31	Lactate ≤ 5.0 mmol/L	137	52
Lactate > 2.44 mmol/L	32	33	Lactate > 5.0 mmol/L	10	12
Total	147	64	Total	147	64

		95% CI			95% CI

Sensitivity	0.52	0.40–0.63	Sensitivity	0.18	0.11–0.29
Specificity	0.78	0.71–0.84	Specificity	0.93	0.88–0.96
PPV	0.51	0.39–0.63	PPV	0.55	0.35–0.74
NPV	0.79	0.71–0.85	NPV	0.72	0.65–0.78

PPV, positive predictive value. NPV, negative predictive value.

**Table 5:** Multiple regression on 30-day mortality (*n* = 211).

Parameter	Coefficient of regression	*p*
Age	−0.027	0.076
Lactate	−0.257	0.001
Oxygen saturation	0.036	0.018
